# A Novel DNAH9 Gene Mutation Causing Primary Ciliary Dyskinesia With an Unusual Association of Jejunal Atresia in a Bahraini Child

**DOI:** 10.7759/cureus.32964

**Published:** 2022-12-26

**Authors:** Hasan M Isa, Fatema A Alkharsi, Maryam Y Busehail, Fayza Haider

**Affiliations:** 1 Department of Pediatrics, Arabian Gulf University, Manama, BHR; 2 Department of Pediatrics, Salmaniya Medical Complex, Manama, BHR; 3 Department of Surgery, Salmaniya Medical Complex, Manama, BHR

**Keywords:** bahrain, jejunal atresia, situs inversus, primary ciliary dyskinesia, kartagener syndrome

## Abstract

Primary ciliary dyskinesia (PCD) is a rare autosomal recessive genetic disorder. It is caused by a defect in the action of the cilia lining multiple organs of the body, including the lungs, the sinuses, hepatobiliary and reproductive organs. In general, the estimated prevalence of this condition is one in 15,000-20,000 individuals. It is characterized by the triad of chronic sinusitis, bronchiectasis, and situs inversus, which occurs in 50% of the cases. It can be associated with other diseases too. Genetic studies can aid in confirming the diagnosis of this condition. A high degree of suspicion about PCD among pediatricians, neonatologists, otorhinolaryngologists, and pulmonologists is essential to make early referrals of patients before they develop irreversible lung damage. Hence, early diagnosis and appropriate treatment are very important. Multicenter collaborations might improve the quality of treatment and patient outcomes. Here, we discuss a case of PCD with a unique association with type IIIb jejunal atresia, and developmental delay secondary to vitamin B12 deficiency. Moreover, the patient was found to have a novel *DNAH9* gene mutation in a compound heterozygous state. This is the first case of this rare disease to be reported from Bahrain. This case report is also associated with an extensive literature review.

## Introduction

Primary ciliary dyskinesia (PCD) is a rare autosomal recessive genetic disorder [1,2]. No sex predilection exists for PCD [3]. In general, the estimated prevalence of the condition is one in 15,000-20,000 individuals [4]. This disease is caused by a defect in the action of the cilia lining the respiratory tract [1,2]. Ciliary functions are vital in multiple organs including the lungs, the sinuses, hepatobiliary and reproductive organs [5]. Deficiency of the dynein arms of the cilia can lead to ciliary immotility in the respiratory tract and fallopian tube, resulting in recurrent chest, ear, nose, throat, and sinus infections, along with infertility [1,6,7]. Moreover, it can lead to a lack of direction and random visceral situs determination resulting in situs inversus or dextrocardia [8]. Subsequently, PCD is characterized by the triad of chronic sinusitis, bronchiectasis, and situs inversus [5]. Situs inversus totalis has been reported in 50% of patients with PCD [8,9].

The diagnosis of PCD relies on classical clinical presentations, radiological findings, and assessment of ciliary functions. Genetic studies can aid in confirming the diagnosis of this condition. Genome analysis has found PCD to be genetically heterogenous [3]. Genes *DNAH5* and *DNA11* on bands 5p15.1 and 9p13.3 respectively are known to cause PCD and both genes encode for dynein [3].

In this report, we present the case of a three-year-old Bahraini male who was diagnosed with PCD, based on the clinical presentation, detection of situs inversus totalis on radiological imaging, and the result of genetic testing that revealed a novel *DNAH9* gene mutation in a compound heterozygous state. Moreover, the patient was found to have an unusual association with type IIIb jejunal atresia. Furthermore, an extensive literature search on this topic was conducted. This is the first case of this rare disease to be reported from Bahrain.

## Case presentation

**Figure 1:**
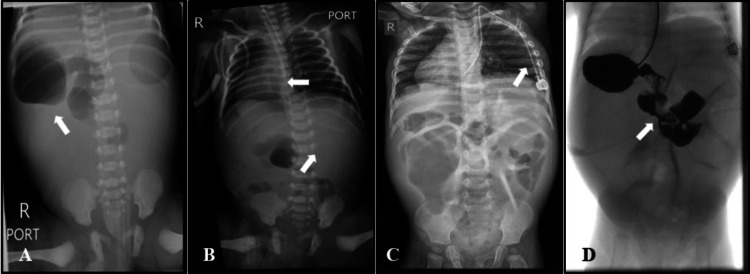
Radiographic imaging 1A: plain abdominal X-ray revealing right-sided gastric shadow; 1B: plain chest and abdominal X-ray showing right-sided cardiac shadow and left-sided hepatic shadow (situs inversus totalis); 1C: postoperative plain chest and abdominal X-ray showing a port-a-cath central line in place and gaseous distension of the bowels; 1D: postoperative Gastrografin study to assess bowel continuity and the total length

The patient was a newborn Bahraini male born after 35 weeks' gestation via a normal vaginal delivery with a birth weight of 2.36 kg. His parents were non-consanguineous, but the mother, her two sisters, and the grandmother had a hearing impairment. His antenatal ultrasound showed dilated bowel loops. Postnatally, the baby gasped and cried at birth; his APGAR score was 9, 10, and 10 at one, five, and 10 minutes, respectively. Abdominal distension associated with erythema and tenderness were noted on examination. A nasogastric tube was inserted, and gastric suction was performed, which obtained 180 ml of greenish fluid. A plain X-ray of the chest and the abdomen showed the cardiac and gastric shadows located on the right side, while the hepatic shadow was on the left side of the abdomen along with signs of intestinal obstruction (Figure 1).

The patient underwent an emergency laparotomy on the first day of life. Intraoperatively, there was a Christmas tree jejunal atresia with an intrauterine gangrenous perforated bowel, which was all amalgamated. The gangrenous bowel was resected along with the atretic segments, and the patient was left with 35 cm of the jejunum and almost 15 cm of the non-used colon. Thus, a jejunostomy was performed and the distal end was brought out as a mucous fistula (colostomy). He was diagnosed to have situs inversus totalis and type IIIb jejunal atresia. A nasogastric tube was inserted and kept on continuous feeds with amino acid-based formula. The patient was stabilized and discharged home with a jejunostomy bag.

Given the presence of situs inversus totalis, PCD was clinically suspected, and clinical exome sequencing was performed at the age of three months. The genetic testing revealed two heterozygous variants of uncertain significance in the *DNAH9* gene in a compound heterozygous state: the *DNAH9* variant c.11086C>T p.(His3696Tyr) causing an amino acid change from His to Tyr at position 3696 and the *DNAH9* variant c.7150G>A p.(Gly2384Arg) causing an amino acid change from Gly to Arg at position 2384. According to SIFT MutationTaster, the *DNAH9* mutation c.11086C>T, p.(His3696Tyr) is disease-causing and the mutation c.7150G>A, p.(Gly2384Arg) is deleterious and disease-causing. Both of these were classified as variants of uncertain significance according to the American College of Medical Genetics and Genomics (ACMG) guidelines [10]. Parental carrier testing was performed, and each one of them was found to be a carrier of one of the mutations, revealing that the mutations were inherited as trans. The patient was the only sibling; therefore, no further segregation analysis was performed. This confirmed the diagnosis of autosomal recessive PCD.

The patient developed recurrent episodes of dumping with diarrhea through the jejunostomy opening. The amount of diarrhea was variable according to the amount of nasogastric tube feeding. Moreover, the patient had occasional vomiting, along with episodes of severe dehydration, metabolic acidosis, and pre-renal azotemia. Therefore, he required frequent admissions to the hospital until the age of 10 months. Nine admissions were due to gastroenteritis, four because of lobar pneumonia, and 10 due to upper respiratory tract infections (URTI). He required multiple courses of intravenous antibiotics.

During the admission, cystic fibrosis (CF) was suspected; a sweat chloride test was performed, and a genetic test for CF transmembrane conductance regulator (CFTR) gene was requested. However, in view of the negative family history of CF, negative sweat chloride test (39 mmol/L; the possibility of CF is considered if the result showed >60 mmol/L), and negative genetic testing for CFTR gene mutation, the diagnosis of CF was ruled out. Port-a-cath was inserted, and total parenteral nutrition (TPN) was given while the patient was in the hospital. Moreover, the child was evaluated by the pulmonologist and had a normal chest examination.

**Figure 2:**
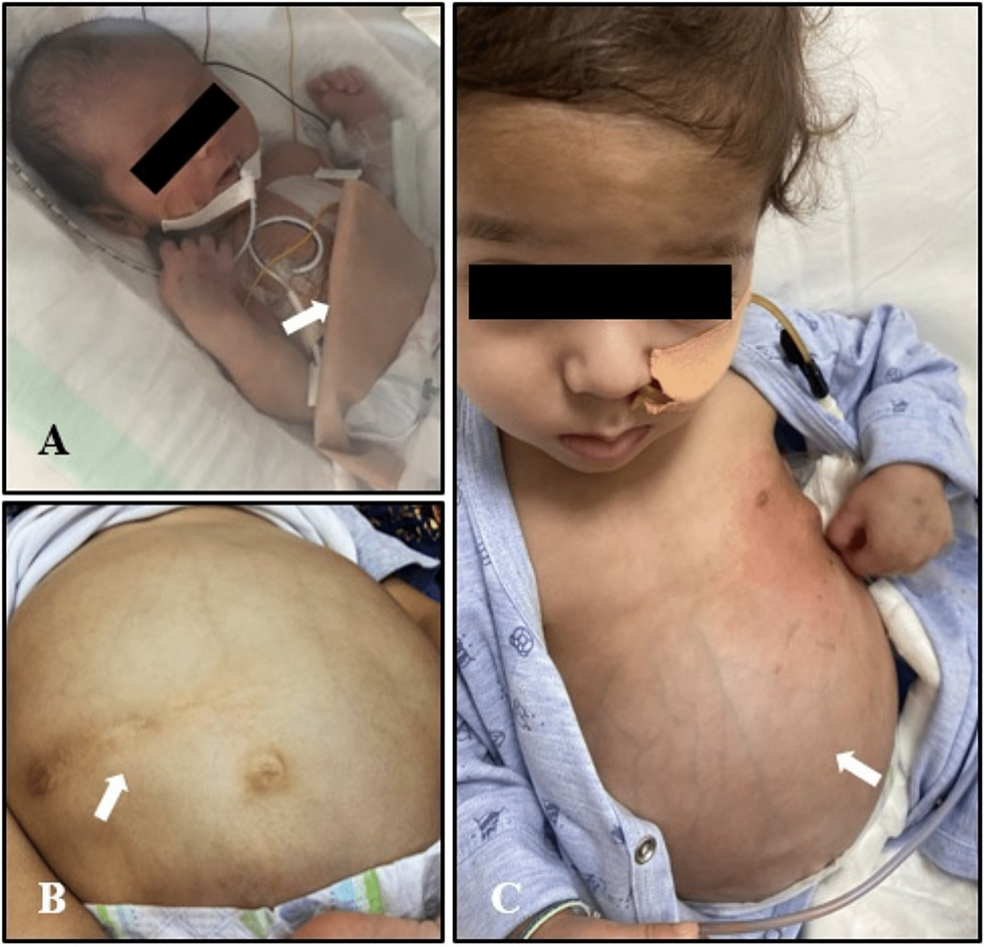
Images of the child at various stages of treatment 2A: a picture of the infant with primary ciliary dyskinesia after resection surgery for jejunal atresia and jejunostomy bag in place; 2B: abdominal scar post-jejunostomy closure; 2C: the infant is on a nasogastric tube for feeding with abdominal distension post-jejunostomy closure Informed consent has been obtained from the patient's parents to publish these pictures

The child had closure of jejunostomy at the age of one year (Figure 2).

Post jejunostomy closure, the stool frequency became less, while the consistency improved. Moreover, the child had a lower gastrointestinal study on the fifth day, which showed a patent anastomosis with no leak or stricture. He started tolerating oral feeds and passing semi-formed stools, and hence he was discharged home.

**Figure 3:**
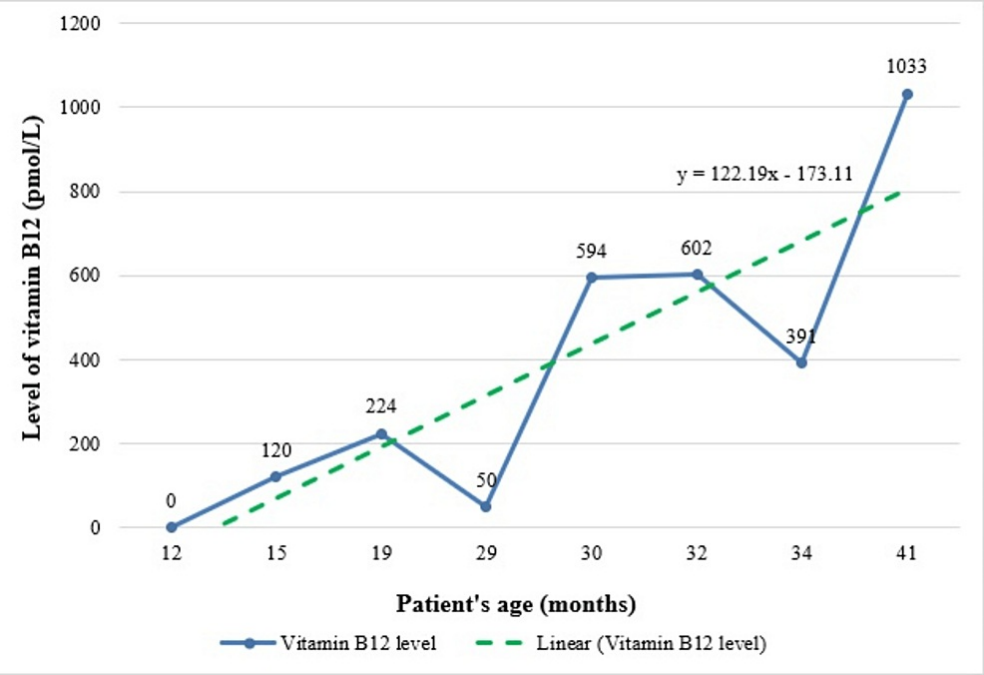
Vitamin B12 levels in the patient after resection surgery for jejunal atresia

At the age of 15 months, the child had an assessment by an otorhinolaryngologist (ENT doctor) and was found to have acute serous otitis media, and required two follow-up visits to the ENT clinic. Moreover, the child was noted to have a gross motor delay. He was able to sit but not stand or walk. The patient was thoroughly investigated and found to have vitamin B12 and vitamin D deficiencies. The level of vitamin B12 was undetectable (normal range: 133-675 pmol/L). Thus, he was started on vitamin B12 1-mg intramuscular injection every four weeks along with vitamin D3 50,000 IU monthly injections. Subsequently, a significant improvement in his condition was observed. Levels of vitamin B12 are shown in Figure 3.

**Figure 4:**
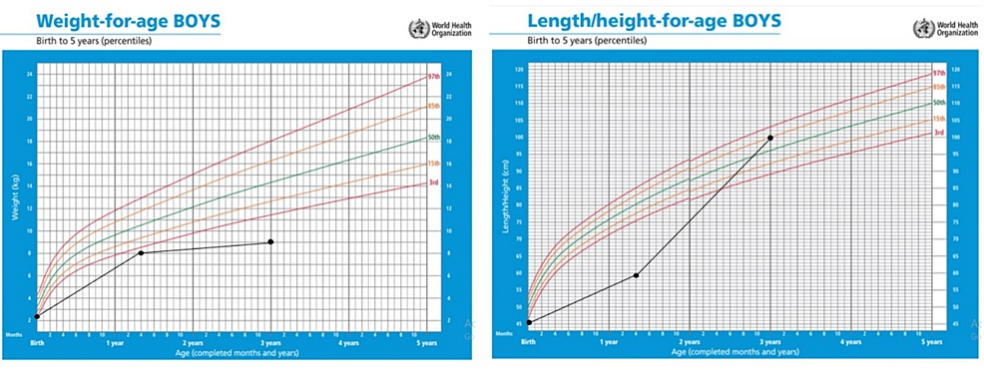
World Health Organization growth charts showing the anthropometric parameters of a child with primary ciliary dyskinesia and type IIIb jejunal atresia

Currently, the patient is three-year-old; he is able to walk and consumes a family diet along with high-caloric milk formula. His weight is 9 kg (<1st percentile) and his height is 100 cm (86th percentile) according to World Health Organization growth parameters (Figure 4).

While the patient has mild abdominal distension, diarrhea and chest symptoms have improved. His current medications are multivitamins, bronchodilators, and oral rehydration salts powder as needed.

## Discussion

We present a case of PCD with an unusual association with type IIIb jejunal atresia and a novel mutation. Upon reviewing the previously reported cases in the past 12 years, a slightly higher prevalence of PCD among males was noted [1,2,5-7,11,12]. Our patient was male, which is in line with the previously reported cases in terms of sex.

The onset of diagnosis differs from one patient to another. Our patient was diagnosed early in life - at the age of three months. Nonetheless, Boon et al. have reported that two-thirds of the patients were diagnosed during childhood with a median age at diagnosis of 5.1 years (interquartile range: 2.0-9.6, range: 0.0-14.9) [13]. Moreover, some patients had a late diagnosis well into adulthood.

Clinical presentation of PCD varies among patients. In our patient, situs inversus totalis, recurrent respiratory tract infections, recurrent episodes of pneumonia, and serous otitis media were the main positive findings. Nonetheless, respiratory tract infections have been found to be one of the most common presentations in patients with PCD, which may appear during the neonatal period and can progress to respiratory insufficiency or failure [14]. However, our patient had a normal chest examination. Recurrent sinus infection is also a common finding in patients with PCD. However, our patient did not develop sinusitis as he was young in age and the sinuses had not yet developed, and sinusitis might appear later in life. Situs inversus (mirror image organ placement) is another important diagnostic criterion and is seen in about 50% of PCD patients [6].

The diagnostic criteria for PCD include a clinical picture suggestive of recurrent chest infections, bronchitis, and rhinitis since childhood, along with one or more of the following: situs inversus, reduced or absent transbronchial mucociliary clearance, cilia showing characteristic ultrastructural defect on electron microscopy, and alive but immotile spermatozoa after puberty in males [6]. In our patient, most of the diagnostic criteria were recorded, but the other symptoms are expected to appear later in life. However, the diagnosis of PCD is challenging and cannot rely on signs and symptoms alone [15]. Our patient was diagnosed early in life at the age of three months. The diagnosis was based on clinical presentation, radiological findings, and genetic testing, which were enough to confirm the diagnosis. Yet, there are many screening tests for PCD, such as exhaled nasal nitric oxide level determination and saccharin test for assessing nasal epithelial mucociliary function. High-speed video microscopy for assessing ciliary beat frequency and pattern, transmission electron microscopic for detecting an ultrastructural ciliary defect, and genetic testing for *DNAI1* and *DNAH5* mutations are confirmatory laboratory tests [16,17].

Abnormal laboratory findings in PCD include reduced nasal nitric oxide level (~10% of normal), reduced ciliary beat frequency (<11 Hz/second), absent ciliary ultrastructure (dynein arms), and mutated *DNAI1 *and *DNAH5* genes [16,17]. Our patient was found to have a novel *DNAH9* gene mutation in a compound heterozygous state. However, most of the reviewed case reports did not involve the genetic testing mentioned, apart from Xu et al.'s study from China where *DNAH5* mutations were detected [18]. The diagnosis in the rest of the articles was established based on clinical presentations and radiological imaging. A correct early diagnosis of PCD can help to significantly avoid unnecessary repeat admissions to hospitals, excessive investigations, and inappropriate antibiotic treatments [5].

PCD can be associated with other diseases such as central giant cell granuloma, extrahepatic biliary atresia, or duodenal atresia [2,12,19]. However, our patient had PCD with jejunal atresia. Upon extensive literature search, we found that no study had reported this association before (Table 1) [1,2,5-7,11,12,18,20].

**Table 1:** Summary of previous studies on patients with primary ciliary dyskinesia *The present study NR: no record

Country	Author, year	Number of patients	Age, years	Sex	Clinical presentation	Associated disease
Bahrain*	Isa et al., 2022	1	0.25	M	Situs inversus and recurrent chest infections	Jejunal atresia
Syria	Ibrahim et al., 2021 [5]	1	3	M	Chronic sinusitis and situs inversus	NR^†^
Turkey	Türkoğlu et al., 2010 [12]	1	15	M	Chronic sinusitis, bronchiectasis, situs inversus	Central giant cell granuloma
Turkey	Uludag Yanaral et al., 2021 [2]	1	1	M	Situs inversus	Extrahepatic biliary atresia
India	Kansal et al., 2012 [1]	1	9	M	Chronic sinusitis, bronchiectasis, situs inversus	NR
India	Mishra et al., 2012 [6]	3	34, 55, 40	M, F, M	Chronic sinusitis, bronchiectasis, situs inversus	NR
India	Gupta et al., 2012 [7]	1	12	M	Chronic sinusitis, bronchiectasis, situs inversus	NR
Ethiopia	Tadesse et al., 2018 [11]	1	24	M	Chronic sinusitis, bronchiectasis, situs inversus	NR
Ethiopia	Hailu et al., 2016 [20]	1	12	F	Chronic sinusitis, bronchiectasis, situs inversus	NR
China	Xu et al., 2016 [18]	2	0.2, 9.5	M, F	Situs inversus in the male patient only	NR

Jejunal atresia is a rare type of small intestinal obstruction that affects newborns and is associated with incomplete formation of a part of the small intestine [21,22]. The prevalence of jejunal atresia has been reported to be between 0.3 and 1.1 per 10,000 births in Europe [23]. However, its prevalence in the Middle East has not been well established. Neonates who were born with jejunal atresia usually present during the first or second day of life with increasing abdominal distention, failure to pass stools, emesis, and feeding problems, which was the case in our patient [21]. Like in our patient, jejunal atresia might be detected even before birth by ultrasound but the diagnosis must be confirmed after birth [22]. To confirm the diagnosis, plain X-rays of the abdomen are needed, which show dilated segments of the bowel filled with gas and liquid [22]. The barium swallow is another method, which may be used to assess the upper digestive tract and confirm the obstruction [22]. In some patients with jejunal atresia, the large bowel may be smaller than normal beyond the point of blockage and called a microcolon, which can be diagnosed by barium enema [22]. In our patient, the antenatal ultrasound showed dilated bowel loops and postnatal radiographs revealed abdominal distension with dilated bowel loops, which suggests intestinal obstruction. Moreover, our patient had a microcolon that was detected intraoperatively. Surgical intervention is the definitive treatment for this condition. Therefore, our patient underwent resection of the atretic and gangrenous segments of the intestine and needed terminal jejunostomy.

This patient developed short bowel syndrome secondary to his jejunal atresia and gangrenous Christmas tree anomaly that required resection. The symptoms of this condition include malnutrition, weight loss, diarrhea, steatorrhea, dehydration, vitamin deficiencies, and electrolyte imbalance [24]. Our patient had severe failure to thrive, along with all of the mentioned symptoms. Therefore, TPN was required, which necessitated hospital admission as the home TPN program is not currently available in Bahrain.

Vitamin B12 is normally absorbed in the last part of the small intestine (ileum) [25]. Accordingly, resection of the terminal ileum can result in vitamin B12 deficiency, as occurred in our patient [25]. Many children are deficient in vitamin B12 even before birth, because their mothers have an undiagnosed vitamin B12 deficiency, and the existing deficiency gets worse when the deficient mother breast-feeds [26]. Moreover, genetic defects like inborn errors of metabolism can also lead to vitamin B12 deficiency [26]. The onset of vitamin B12 deficiency symptoms occurs more rapidly in infants, including developmental delay, hypotonia, tremor, seizures, failure to thrive, reduced intelligence quotient, and mental retardation [26]. Children with vitamin B12 deficiency exhibit speech, language, and social delays, behavioral issues, and problems with fine and gross motor movement [26]. Treating this disorder with vitamin B12 supplementation can result in a rapid improvement of the condition [26]. This was the case with our patient who responded rapidly to vitamin B12 injections and started to stand and walk.

PCD is a genetic disease and has no definitive treatment. Treatments for these patients are mainly symptomatic treatments that include intermittent or constant oral or intravenous administration of antibiotics to treat respiratory infections, inhaled bronchodilators, mucolytics, oral corticosteroids, and chest physiotherapy for bronchiectasis and pneumonia [27]. Administration of the annual influenza vaccine and regular doses of the pneumococcus vaccine as a part of the childhood immunization program is also necessary to prevent frequent infections [27].

Our patient is still wasted and stunted, and he needs proper nutritional support. The prognosis of this child is dependent on his nutritional status as a good nutritional status is associated with a good prognosis and vice versa.

## Conclusions

PCD is an uncommon genetic disease. The association of PCD with jejunal atresia is extremely rare. Detection of a novel *DNAH9 *gene mutation in a compound heterozygous state causing PCD is particularly important for the diagnosis of this condition. Early diagnosis and appropriate management of the associated complications of PCD can help to significantly avoid unnecessary repeat hospital admissions, excessive investigations, and inappropriate antibiotic use. A high degree of suspicion about PCD among pediatricians, neonatologists, ENT surgeons, and pulmonologists is very important, to make early referrals of patients before they develop irreversible lung damage. Multicenter collaborations might improve the quality of treatment and patient outcomes.
